# Optimal Design of Large Aperture Primary Mirror of Spaceborne Solar EUV Imager Based on Optomechanical Coupling Analysis

**DOI:** 10.3390/s26175652

**Published:** 2026-09-05

**Authors:** Bin Huang, Xinkai Li, Zhaohui Li, Kefei Song

**Affiliations:** Changchun Institute of Optics, Fine Mechanics and Physics, Chinese Academy of Sciences, Changchun 130031, China; huangbin@ciomp.ac.cn (B.H.);

**Keywords:** spaceborne solar EUV imager, optomechanical coupling analysis, large aperture primary mirror, dimensional optimization, sensitivity analysis

## Abstract

This paper proposes a parametric modeling and multi-objective optimization method based on optomechanical coupling analysis to address the design optimization of large-aperture primary mirrors in space-based solar extreme ultraviolet (EUV) imaging instruments. By establishing a parametric model of the primary mirror and combining topology optimization with dimensional optimization, the influence of key parameters—including mirror thickness, rib width, and lightweight hole dimensions—on the mirror surface shape accuracy (RMS) and structural fundamental frequency is systematically analyzed. The study employs finite element simulation and optomechanical coupling data processing algorithms to extract the rigid-body displacement and surface shape error of the primary mirror under gravitational loading and identifies high-impact parameters through sensitivity analysis. After optimization, the RMS values of the mirror’s surface shape in all three directions under a 1 g gravitational load are all better than 4.5 nm, meeting the requirements for spaceborne payloads. Experimental validation shows that the surface shape RMS of the assembled primary mirror is only 0.022λ (with λ = 632.8 nm), and the system wavefront error is less than 0.08λ, significantly surpassing the design specification requirement of 0.1λ.

## 1. Introduction

The changes in space weather have a decisive influence on satellites, communications systems, the electric grid, aviation workers and passengers, and even astronauts, who are exposed to solar storm radiation while living and working on the International Space Station. Moreover, space weather has critical effects on Earth weather, leading to high-impact environmental phenomena such as severe storms, hurricanes, fires, and volcanic eruptions across the world. The main cause of changes in space weather is the variation in activities on the surface of the Sun, including flares and coronal mass ejections and so forth. Therefore, solar observation is of great significance for the early warning of emergency space weather and natural disasters.

Space-based solar EUV (Extreme Ultraviolet) imagers are used for real-time monitoring of the activity status on the surface of the Sun in orbit. Currently, researchers around the world are engaged in the development of solar EUV imagers. Solar EUV imagers usually adopt a coaxial two-reflector Cassegrain optical system. To improve the resolution and signal-to-noise ratio of the system, the primary mirror component is designed to have a large aperture, and the structural design optimization of the large-aperture primary mirror and the design optimization of its supporting structure are one of the key technologies. Numerous optimization solutions have been proposed among researchers all over the world concerning the stiffness of the large aperture primary mirror and the working performance of optical systems. J. M. Cheng studied the mechanical responses of a Bessel-point supporting thin mirror with a downward working surface to reduce the gravitational deformation and improve the surface accuracy, and the obtained deformation was reduced by 30.86% [1]. Andre V. Santos presented a method to optimize the position of primary mirrors with the goal of producing a more continuous primary optic, eventually closer to the theoretical limits of solar concentration [2].

According to the optomechanical coupling analysis theory, the surface shape error can be acquired by finite element analysis results, and the rigid-body displacement of the mirror needs to be subtracted. Some research with respect to post-processing algorithm results of finite element analysis has been carried out. H. S. Yang presented an algorithm based on a constrained optimization method to accomplish the parameter extraction of large rigid-body displacement [3]. G. Z. Xu presented a high-precision algorithm based on a comprehensive surface parameter to evaluate the primary mirror change under the external loads. The discrete error of finite element discretization of optical surfaces is addressed and the elimination of discrete error is discussed as well [4]. Moreover, some more research is demonstrated with regard to lightweight design and optimal support design of large-aperture mirrors in space cameras. The performance and specifications are guaranteed under the external dynamic and thermal conditions by a high ratio of stiffness to mass design [5,6,7,8].

Besides the optimal design of primary mirror based on optomechanical coupling analysis, some focus is also concentrated on optimization algorithms and methods, including topology and dimensional optimization. X. D. Wang established a floating projection topology optimization method with a p-norm function to realize stress minimization, and some optimization problems based on stress-constraint can be reformulated by introducing a global measure of an optimized design, the average solid stress [9]. Z. Fan proposed an extending bi-directional evolutionary structural optimization (BESO) method for compliance minimization design subject to both constraints on volume fraction and maximum von Mises stress [10]. Maximilian Eckrich proposed a topology optimization procedure that allows for unidirectional but also biaxial fiber orientations based on the stress state within the finite elements. The stress state at fiber intersections is improved by reducing the probability of inter-fiber failure [11]. Alexander A. Safonov discussed a method which is used to optimize a topology of 3D continuous fiber-reinforced additively manufactured structures and makes it possible to simultaneously search for density distribution and local reinforcement layup in 3D composite structures of transversely isotropic materials [12]. Topology optimization and some relative algorithms are employed as well in the design of compliant mechanisms [13], conduction and transfer of heat [14,15], truss-structures design [16,17] and multi-objective structure optimization [18,19,20].

Even though lots of algorithms and solutions with respect to optimization of the primary mirror are proposed, little research is focused on primary mirrors with large apertures, especially primary mirrors in EUV solar observation. Therefore, to realize the optimal design of the primary mirror, parametric modeling of the primary mirror, dimensional optimization, and sensitivity analysis are accomplished in this paper. The remainder of this paper is organized as follows. In Section 2, parametric models of the primary mirror are established and data processing algorithms of optomechanical coupling analysis are deduced. In Section 3, a numerical simulation model and results of primary mirror assembly are obtained, including topology optimization and dimension optimization. In Section 4, surface shape interferogram of the primary mirror is given and wave-front aberration results of the optical system are acquired. Finally, a conclusion is drawn in Section 5.

## 2. Modeling and Formulation

### 2.1. Parametric Modeling of Primary Mirror

According to the result of optical design, the clear aperture of the primary mirror is 205 mm. The mechanical diameter of the primary mirror is determined to be 210 mm when the machining technology is taken into account. The surface of the primary mirror is parabolic and the aspherical equation is as follows.

(1) $$x=\frac{cy^{2}}{1+\sqrt{1-(K+1)c^{2}y^{2}}}$$

where

c = 1/R, R is the radius of curvature;
K is the conic coefficient.

**Figure 1:**
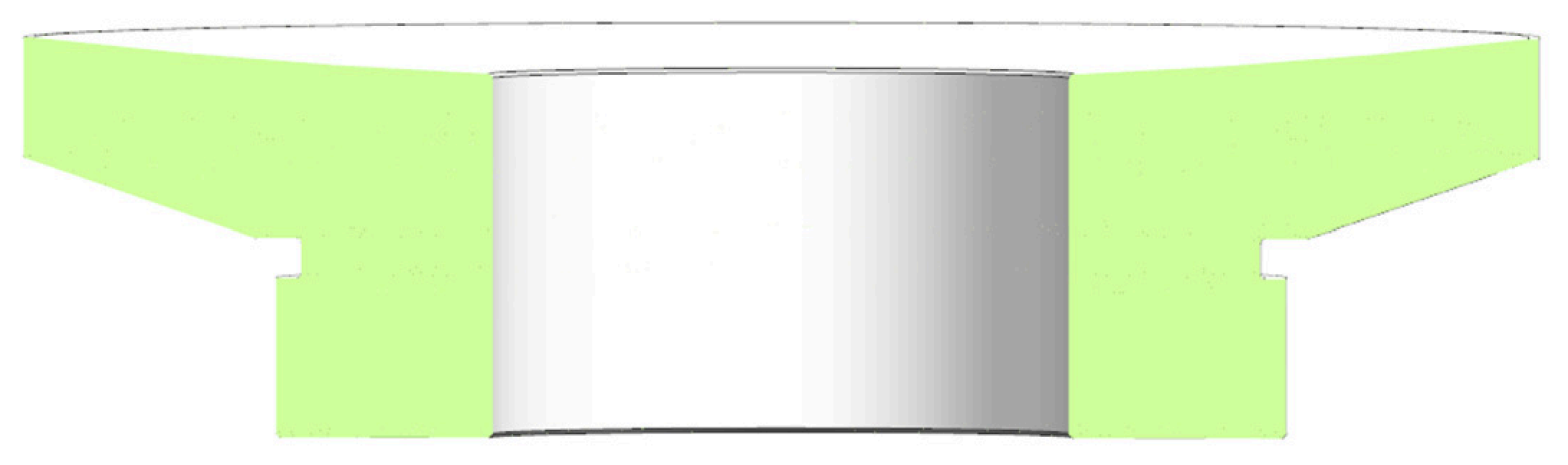
Outline of primary mirror.

The support structure forms of the primary mirror mainly include four types: toroidal support, radial support, central support, and back support. Based on the dimensions and structural form of the primary mirror in the EUV imager, and by comparing the characteristics of various support schemes, it is finally determined that the primary mirror adopts a central support structure. To adjust the center of mass of the primary mirror as far back as possible from the reflective surface, a stepped cylindrical structure is designed at the rear of the reflective surface. The outline of the primary mirror is as shown in Figure 1.

**Figure 2:**
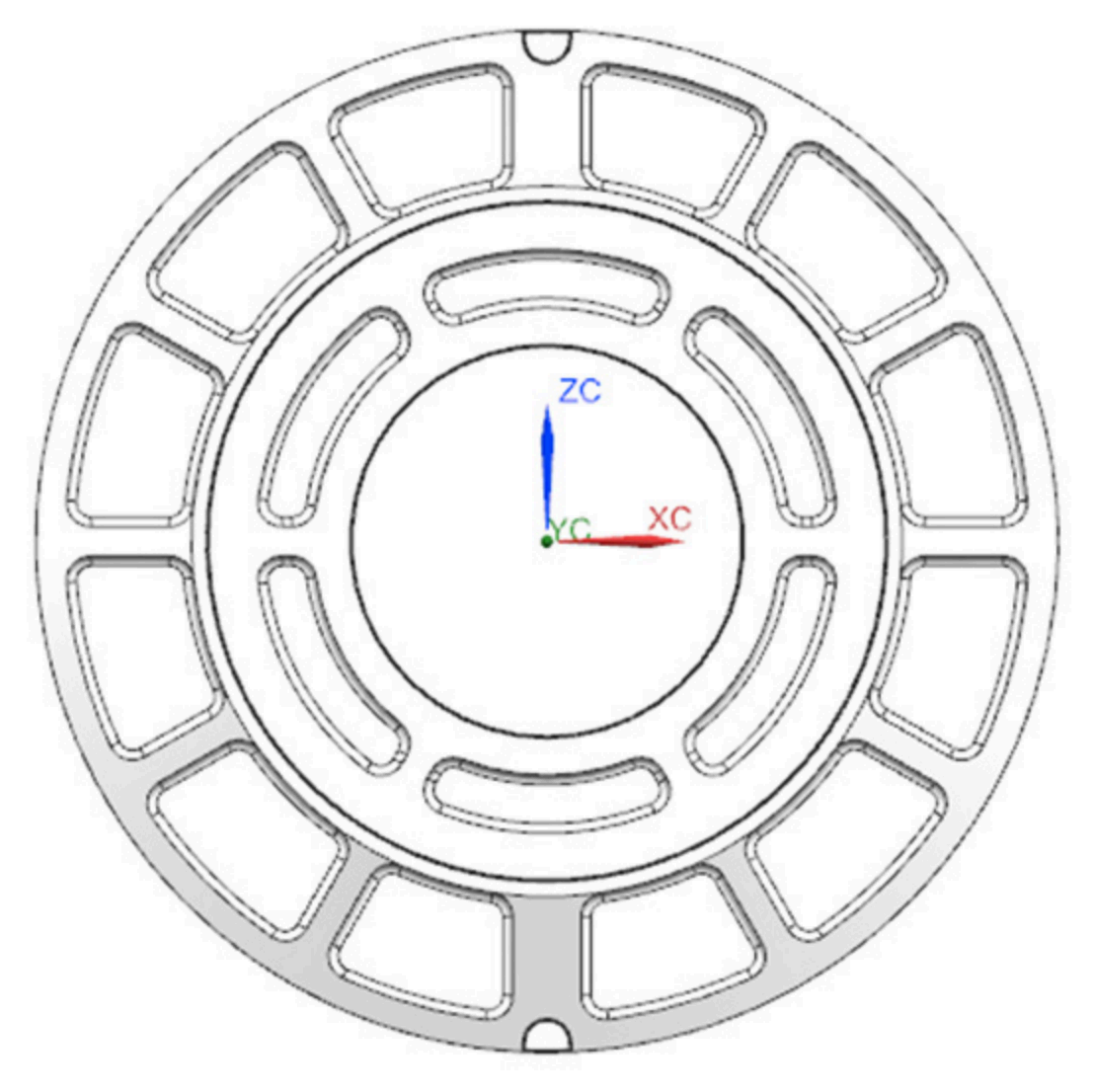
Structure after lightweight design.

The light weight of the primary mirror is also of great importance. For the light weight of the primary mirror, commonly used lightweight forms include triangular holes, quadrilateral holes, hexagonal holes, and sector holes. Among these, circular holes feature high stiffness but a low lightweight rate, while sector holes are mainly used in mirrors with central holes. Considering the dimensions, structural form, and support form of the primary mirror, the lightweight form of sector holes is adopted. The preliminary structure after lightweight design is as shown in Figure 2.

**Figure 3:**
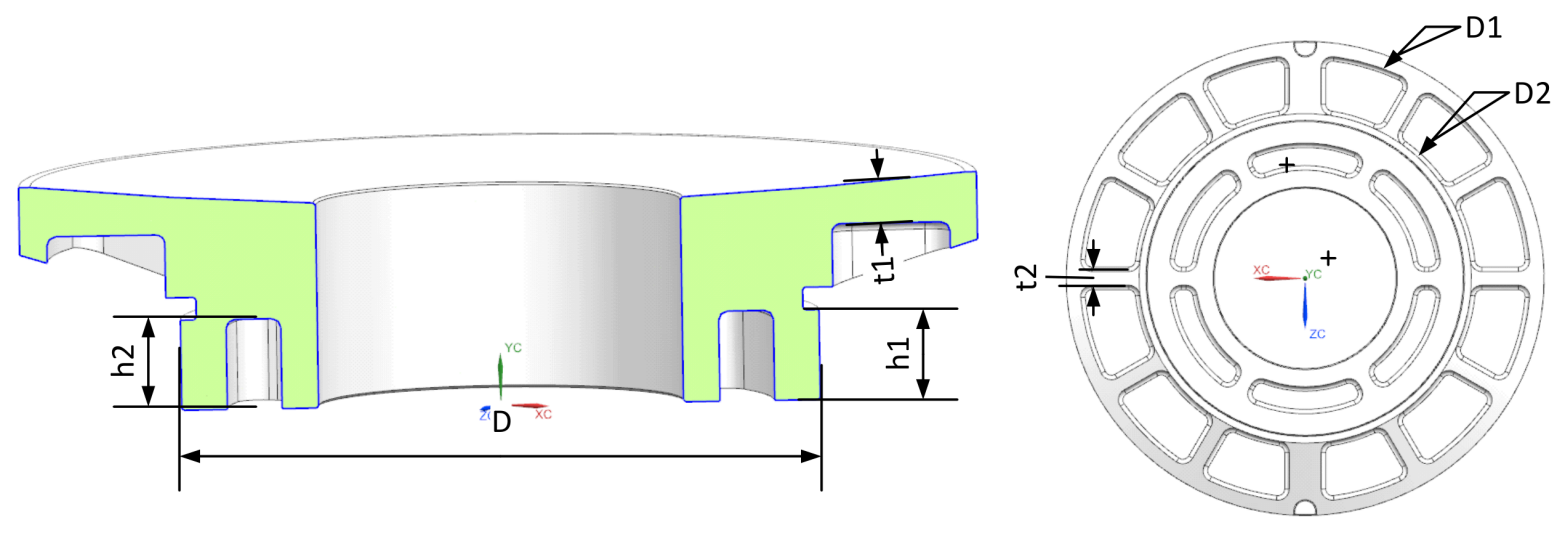
Parametric model of primary mirror.

Moreover, a parametric model of the primary mirror is established. The key design parameters of the primary mirror mainly include mirror surface thickness, rib width, and other parameters, as shown in Figure 3.

Where

t1: thickness of the mirror surface;
t2: width of ribs;
D1: outer diameter;
D2: inner diameter;
h1: height of the rear cylinder;
h2: depth of lightweight;
D: diameter of rear cylinder.

Based on the above parameters, the constraints and objective function for the optimal design of the primary mirror are established as follows.

$$\min P_{RMS}=f(t1,t2,D1,D2,h1,h2,D)\;\mathrm{subject}\;\mathrm{to}\;m_{0}\geq m=g(t1,t2,D1,D2,h1,h2,D)$$

where

*P*_RMS_ is the surface shape RMS of the mirror;
*m* is the actual mass of the mirror;
*m*_0_ is the upper limit of the mirror mass.

### 2.2. Data Processing Algorithms of Optomechanical Coupling Analysis

To analyze the influence of the above-mentioned design parameters on the objective function, it is necessary to establish a finite element model of the primary mirror and connecting ring assembly and analyze the variation in the surface figure of the primary mirror’s optical surface under typical operating conditions. By examining the corresponding relationship between each set of parameters and the surface figure, the sensitivity of each parameter with respect to the objective function can be obtained, thereby enabling in-depth design optimization of highly sensitive parameters.

On the other hand, the results of the finite element analysis of the primary mirror’s optical surface do not directly represent the surface figure; instead, they are comprehensive outcomes that include both surface rigid displacement and surface distortion. Therefore, it is necessary to extract and remove the surface rigid displacement. After processing the remaining distortion data, the surface figure peak-to-valley (PV) value and root mean square (RMS) value of the optical surface can be obtained.

In the finite element model, a global coordinate system is established at the vertex of the primary mirror. Given the initial node coordinates of the primary mirror’s optical surface as $\left(x_{0i},y_{0i},z_{0i}\right)$, the node coordinates after pure rigid displacement as $\left(x_{1i},y_{1i},z_{1i}\right)$, and the comprehensive node coordinates with surface distortion as $\left(x_{2i},y_{2i},z_{2i}\right)$, without loss of generality, assume that the rigid displacement of the optical surface is $\left(a,b,c,\theta_{x},\theta_{y},\theta_{z}\right)$, and then we have

$$\left[\begin{array}{c} x_{1}\\ y_{1}\\ z_{1}\\ 1 \end{array}\right]=\left[\begin{array}{cccc} 1 & -\theta_{z} & \theta_{y} & a\\ \theta_{z} & 1 & -\theta_{x} & b\\ -\theta_{y} & \theta_{x} & 1 & c\\ 0 & 0 & 0 & 1 \end{array}\right]\left[\begin{array}{c} x_{0}\\ y_{0}\\ z_{0}\\ 1 \end{array}\right]$$

$$\left\{\begin{array}{c} x_{1}=x_{0}-y_{0}\theta_{z}+z_{0}\theta_{y}+a\\ y_{1}=x_{0}\theta_{z}+y_{0}-z_{0}\theta_{x}+b\\ z_{1}=-x_{0}\theta_{y}+y_{0}\theta_{x}+z_{0}+c \end{array}\right.$$

The least squares objective function for the nodes on the optical surface before and after deformation is established as follows.

$$W=\sum \left[{\left(x_{2i}-x_{1i}\right)}^{2}+{\left(y_{2i}-y_{1i}\right)}^{2}+{\left(z_{2i}-z_{1i}\right)}^{2}\right]$$

This objective function has a total of 6 variables. By taking the partial derivatives with respect to the above variables and setting the derivatives equal to zero, the objective function attains an extremum. Therefore, we have

$$\begin{array}{c} \frac{\partial W}{\partial a}=0,\;\sum x_{0i}-\theta_{z}\sum y_{0i}+\theta_{y}\sum z_{0i}+\sum a=\sum x_{2i}\\ \frac{\partial W}{\partial b}=0,\theta_{z}\sum x_{0i}+\sum y_{0i}-\theta_{x}\sum z_{0i}+\sum b=\sum y_{2i}\\ \frac{\partial W}{\partial c}=0,-\theta_{y}\sum x_{0i}+\theta_{x}\sum y_{0i}+\sum z_{0i}+\sum c=\sum z_{2i}\\ \frac{\partial W}{\partial \theta_{x}}=0,-\theta_{x}\sum \left(y_{0i}^{2}+z_{0i}^{2}\right)+\theta_{y}\sum x_{0i}y_{0i}+\theta_{z}\sum x_{0i}z_{0i}+b\sum z_{0i}-c\sum y_{0i}=\sum y_{2i}z_{0i}-y_{0i}z_{2i}\\ \frac{\partial W}{\partial \theta_{y}}=0,\theta_{x}\sum x_{0i}y_{0i}-\theta_{y}\sum \left(x_{0i}^{2}+z_{0i}^{2}\right)+\theta_{z}\sum y_{0i}z_{0i}-a\sum z_{0i}+c\sum x_{0i}=\sum z_{2i}x_{0i}-z_{0i}x_{2i}\\ \frac{\partial W}{\partial \theta_{z}}=0,\theta_{x}\sum x_{0i}z_{0i}+\theta_{y}\sum y_{0i}z_{0i}-\theta_{z}\sum \left(x_{0i}^{2}+y_{0i}^{2}\right)+a\sum y_{0i}-b\sum x_{0i}=\sum x_{2i}y_{0i}-x_{0i}y_{2i} \end{array}$$

The above system of equations is organized into a matrix form below.

$$\left[\begin{array}{cccccc} \sum 1 & 0 & 0 & 0 & \sum z_{0i} & -\sum y_{0i}\\ 0 & \sum 1 & 0 & -\sum z_{0i} & 0 & \sum x_{0i}\\ 0 & 0 & \sum 1 & \sum y_{0i} & -\sum x_{0i} & 0\\ 0 & \sum z_{0i} & -\sum y_{0i} & -\sum \left(y_{0i}^{2}+z_{0i}^{2}\right) & \sum x_{0i}y_{0i} & \sum x_{0i}z_{0i}\\ -\sum z_{0i} & 0 & \sum x_{0i} & \sum x_{0i}y_{0i} & -\sum \left(x_{0i}^{2}+z_{0i}^{2}\right) & \sum y_{0i}z_{0i}\\ \sum y_{0i} & -\sum x_{0i} & 0 & \sum x_{0i}z_{0i} & \sum y_{0i}z_{0i} & -\sum \left(y_{0i}^{2}+x_{0i}^{2}\right) \end{array}\right]\left[\begin{array}{c} a\\ b\\ c\\ \theta_{x}\\ \theta_{y}\\ \theta_{z} \end{array}\right]=\left[\begin{array}{c} \sum \left(x_{2i}-x_{0i}\right)\\ \sum \left(y_{2i}-y_{0i}\right)\\ \sum \left(z_{2i}-z_{0i}\right)\\ \sum \left(y_{2i}z_{0i}-z_{2i}y_{0i}\right)\\ \sum \left(z_{2i}x_{0i}-x_{2i}z_{0i}\right)\\ \sum \left(x_{2i}y_{0i}-y_{2i}x_{0i}\right) \end{array}\right]$$

After solving for the rigid displacement, the nodal normal displacement corresponding to the optical surface distortion can then be determined, namely $\left(\delta x_{i},\delta y_{i},\delta z_{i}\right)$

$$\left\{\begin{array}{c} \delta x_{i}=x_{2i}-x_{1i}\\ \delta y_{i}=y_{2i}-y_{1i}\\ \delta z_{i}=z_{2i}-z_{1i} \end{array}\right.$$

Without loss of generality, assuming that the Z-axis of the primary mirror coordinate system corresponds to the optical axis, the calculation formulas for the RMS and PV values of the primary mirror’s surface figure are as follows.

$$\left\{\begin{array}{c} RMS=\sqrt{\frac{1}{N}\sum \delta z_{i}^{2}}\\ PV=\max(\delta z_{i})-\min(\delta z_{i}) \end{array}\right.$$

Using the above formulas, given the nodal coordinates of the surface before and after deformation, the rigid displacement and surface figure of any deformed surface can be determined.

## 3. Numerical Simulation

### 3.1. Topology Optimization

Before the detailed design of the primary mirror, the topological structure of the primary mirror is first optimized, and a mathematical model for topology optimization is established. The optimization model is presented as follows.

$$\;\;\;\;\;\;\;\;\mathrm{Objective}\;\mathrm{function}\;=\;\min\;C\;\mathrm{subject}\;\mathrm{to}\left\{\begin{array}{c} m\leq m_{0}\\ f\geq f_{0}\\ disp\leq disp_{0} \end{array}\right.$$

where

*C* is the compliance of the mirror structure;
*m* is the mass of the mirror and *m*_0_ is the allowable maximum;
*f* is the first frequency of the mirror and *f*_0_ is the allowable minimum;
*Disp* is the displacement of the mirror and *disp*_0_ is the allowable maximum.

**Figure 4:**
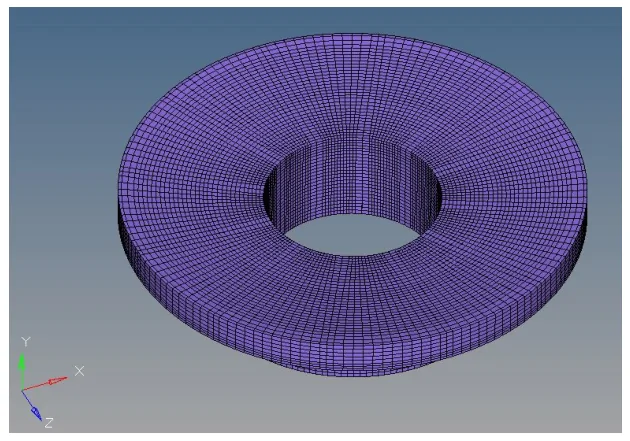
Finite element mesh model.

A topological optimization model of the primary mirror was established in HyperMesh [21], with the finite element mesh model as shown in Figure 4.

Considering the characteristics of the primary mirror such as stiffness and coefficient of thermal expansion (CTE), the primary mirror is made of microcrystalline glass. Meanwhile, to match the CTE of the primary mirror, the connecting ring is made of invar steel. Relevant material property parameters are as shown in Table 1.

**Figure 5:**
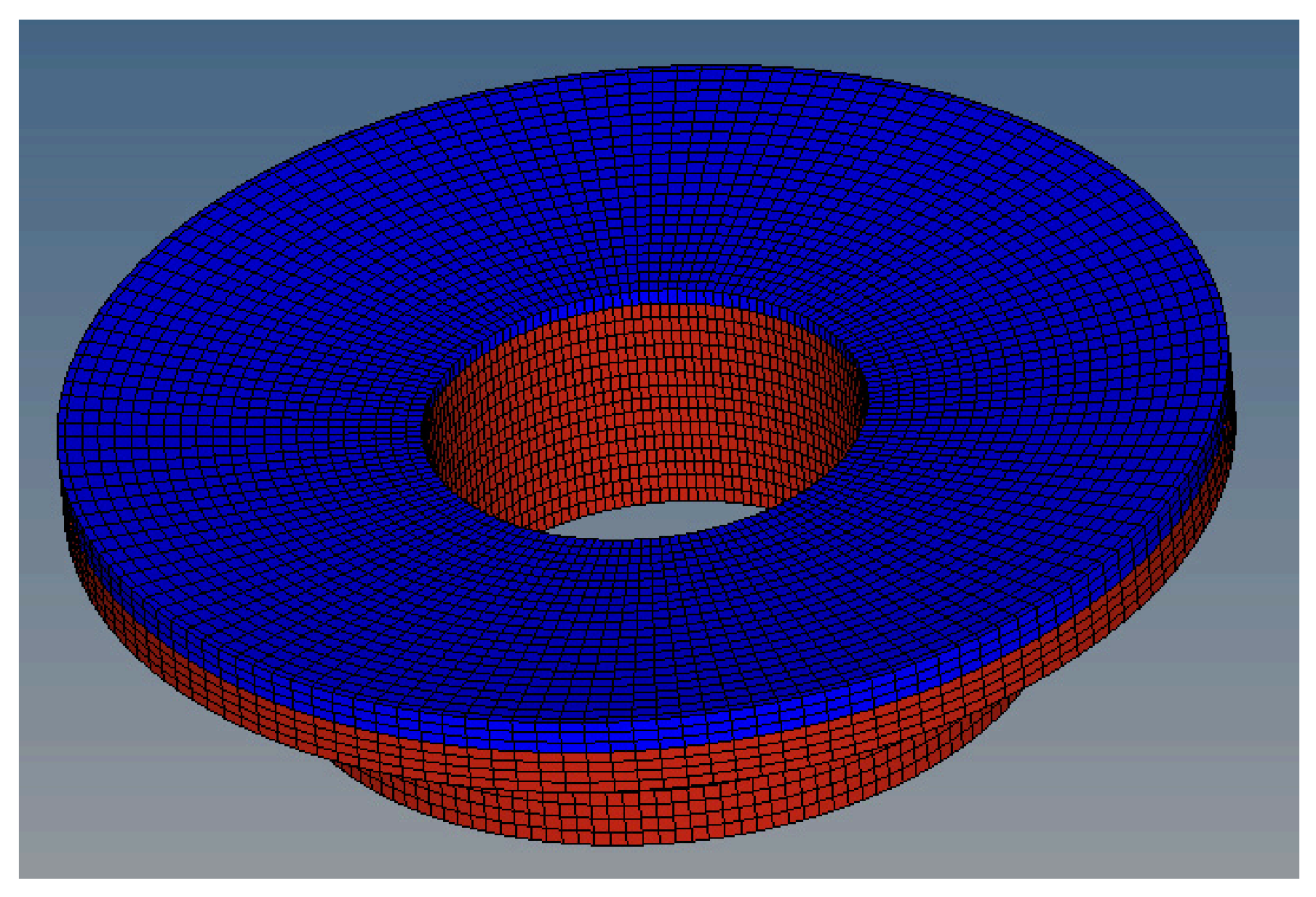
Optimization regions setup.

In the finite element model, the optimization regions and non-optimization regions are clearly defined. In the primary mirror structure, the mesh of the upper surface is designated as the non-optimization region (corresponding to the blue mesh), while the remaining meshes are designated as the optimization regions (corresponding to the redmesh), with the setup results as shown in Figure 5.

**Figure 6:**
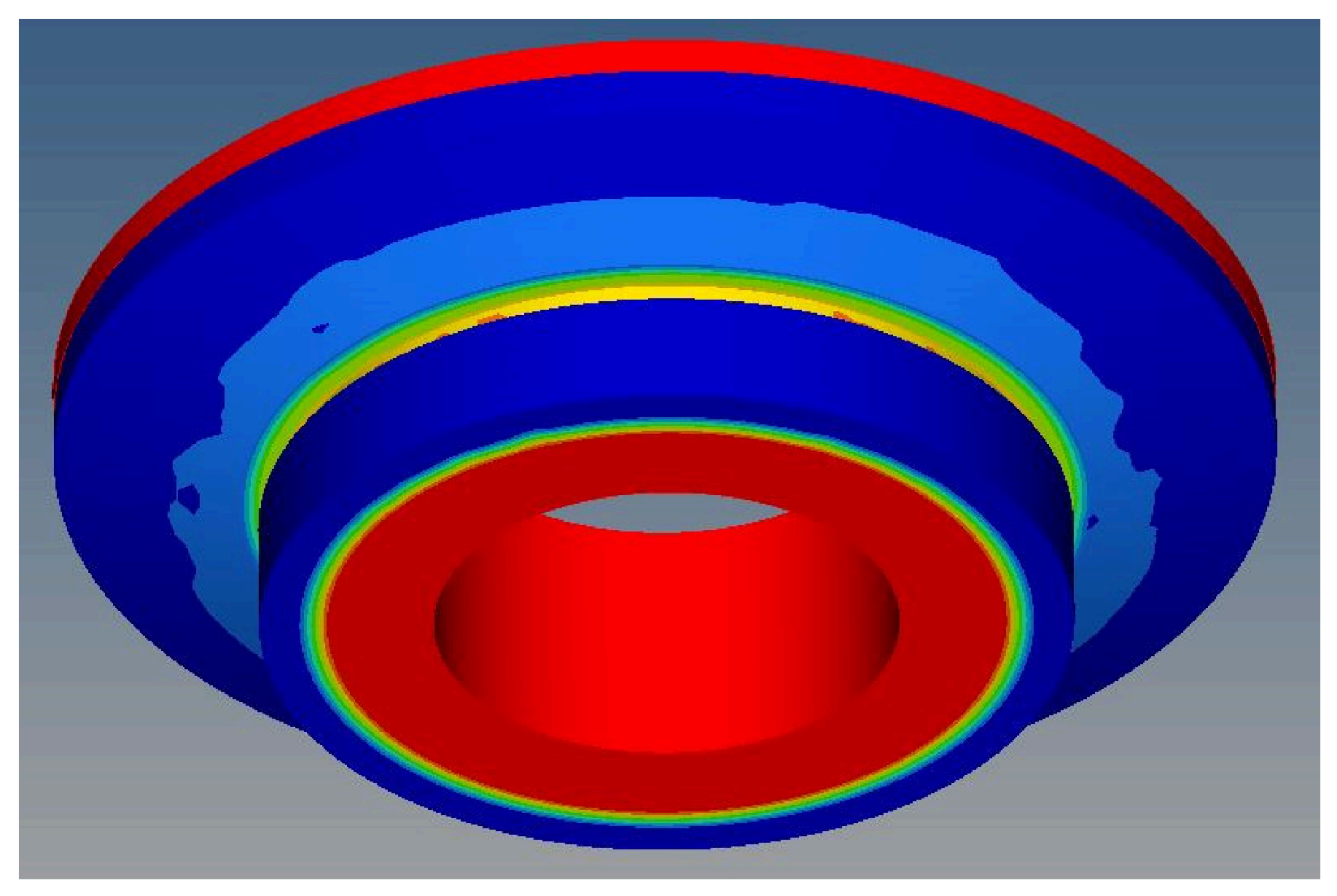
Topology optimization result.

The topological optimization results of the primary mirror are as shown in Figure 6. In the figure, the blue regions contribute less to the primary mirror’s performance and can be removed during detailed structural design; the red regions contribute more significantly to the primary mirror’s performance and need to be retained during detailed structural design.

**Figure 7:**
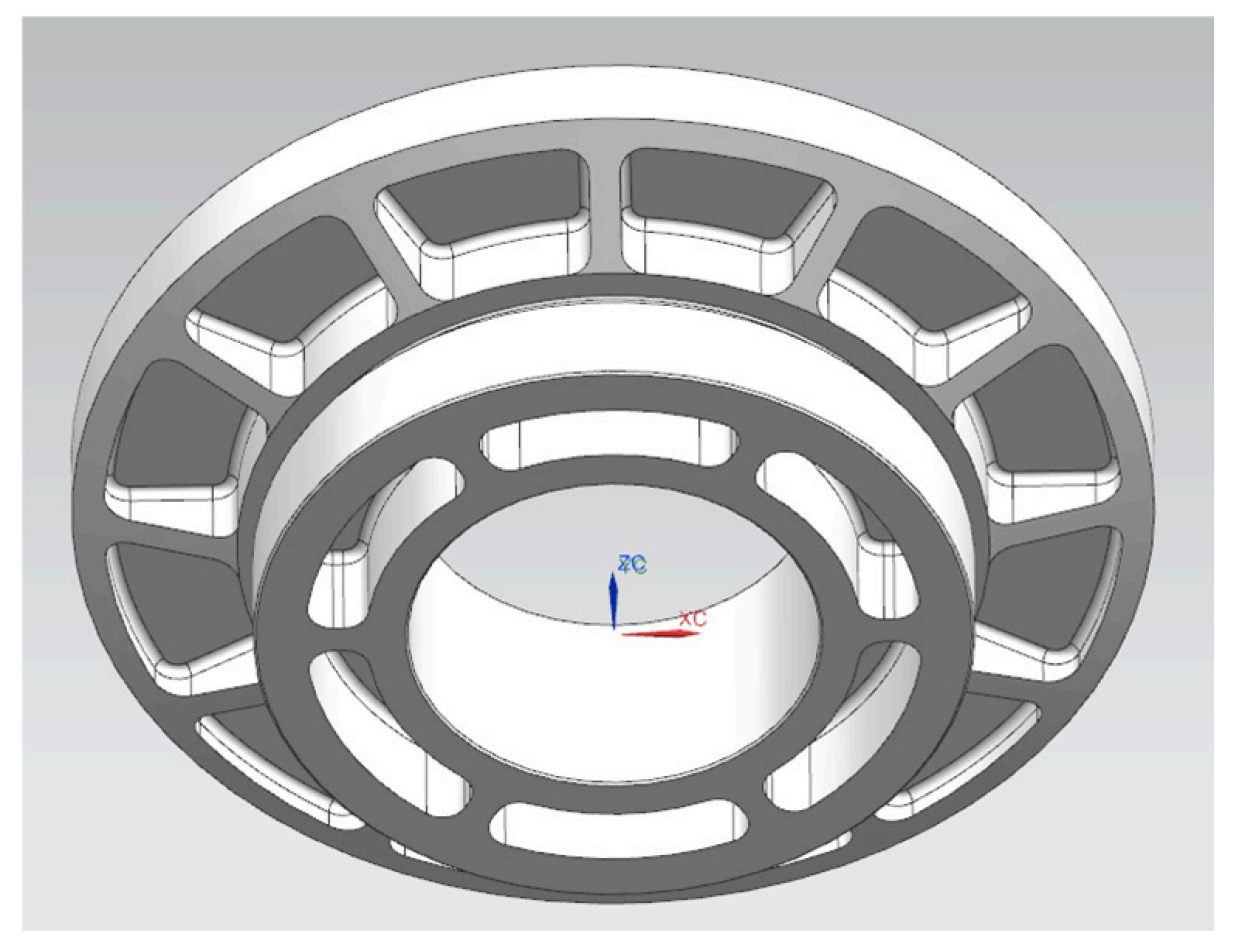
Preliminary structural form of mirror.

Taking into account the primary mirror’s manufacturing process, common lightweight styles, and its performance requirements, the preliminary structural form of the finally topologically optimized primary mirror is as shown in Figure 7.

### 3.2. Dimensional Optimization

#### 3.2.1. Dataflow Setup

**Figure 8:**
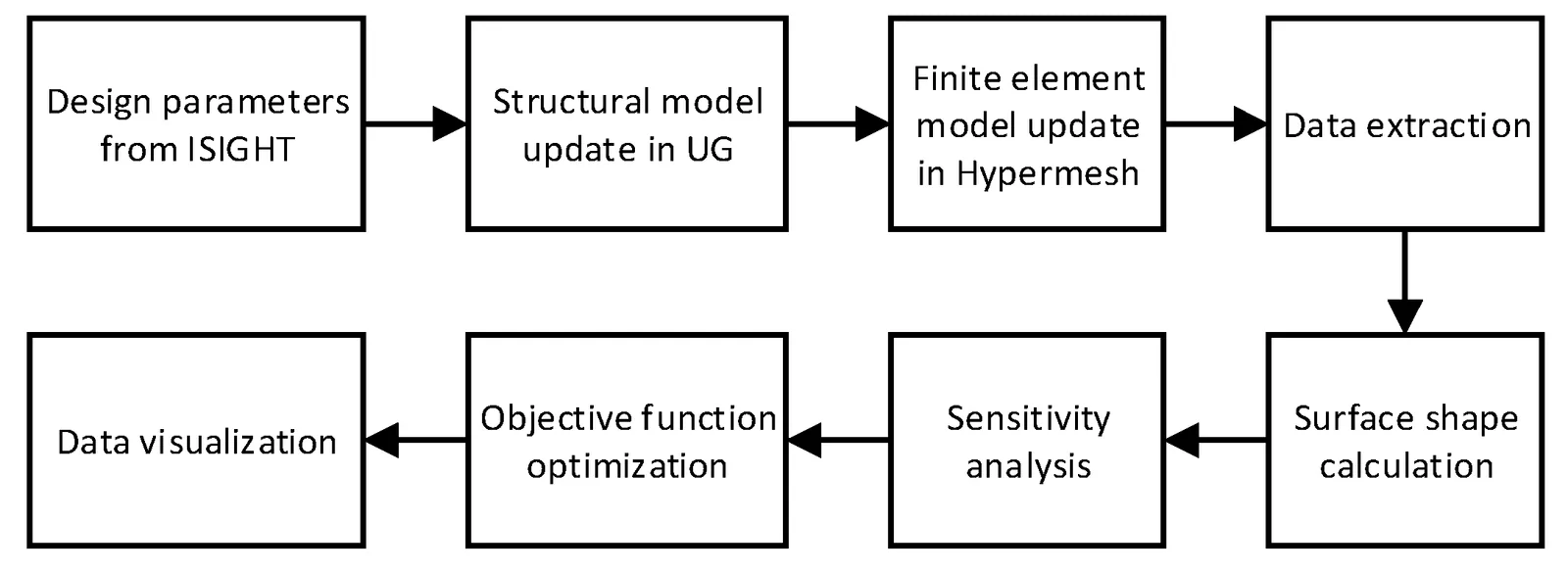
Analysis and optimization process.

To analyze the influence of each structural parameter of the primary mirror on its weight and surface figure, it is necessary to establish a design optimization process that integrates automatic parameter modeling, finite element analysis (FEA), parameter sensitivity analysis, objective optimization, and data visualization. This requires an analysis and optimization platform capable of being compatible with multiple engineering software. As a multi-platform analysis and optimization software, ISIGHT version 2016 has been widely applied in the fields of optomechanical–thermal co-simulation and multi-field coupling analysis. For the primary mirror design optimization of the solar EUV imager, the analysis and optimization process is as shown in Figure 8.

**Figure 9:**
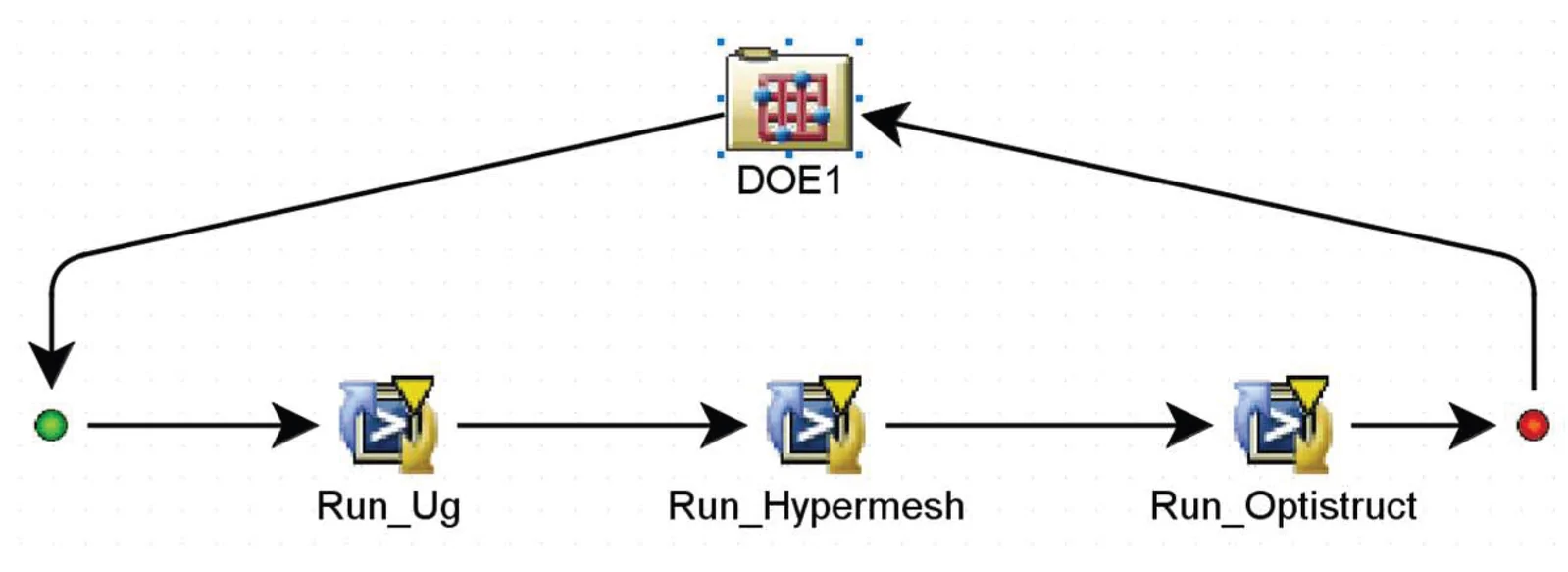
Simulation data flow diagram.

Based on the above block diagram, a multi-platform data flow simulation process based on automatic model updating is established in ISIGHT, and the final simulation data flow diagram is as shown in Figure 9.

#### 3.2.2. Sensitivity Analysis

The dataflow comprises seven input parameters and two output parameters. An initial round of simulation is conducted to determine the sensitivity of each input parameter relative to the output parameters. After identifying the highly sensitive input parameters, further simulations are performed within narrowed numerical ranges for these relevant parameters to establish the relationship between the inputs and outputs. This process ultimately leads to the determination of the key dimensions for the primary mirror. The input and output parameters are listed in Table 2.

**Figure 10:**
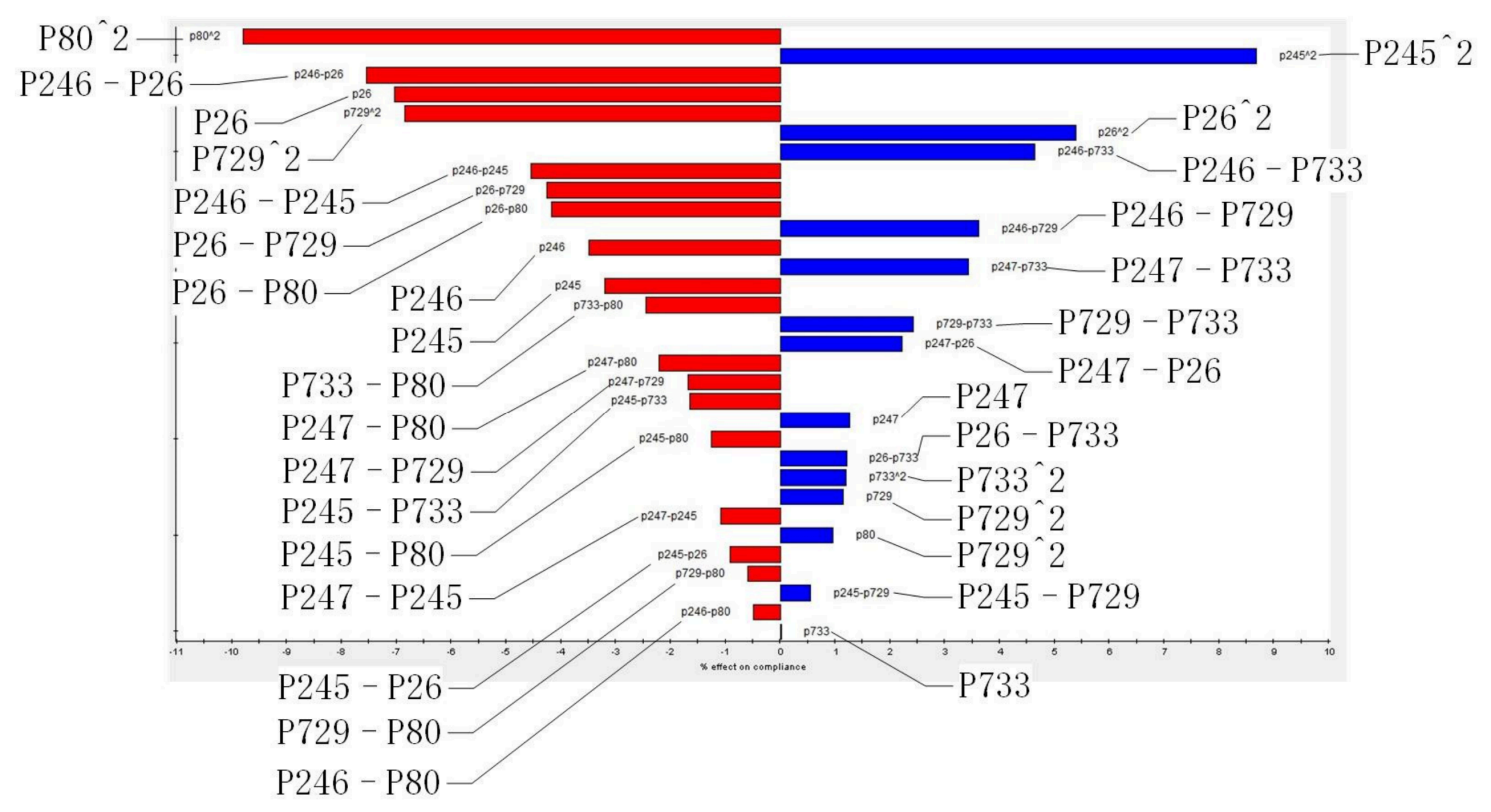
Pareto result of compliance.

**Figure 11:**
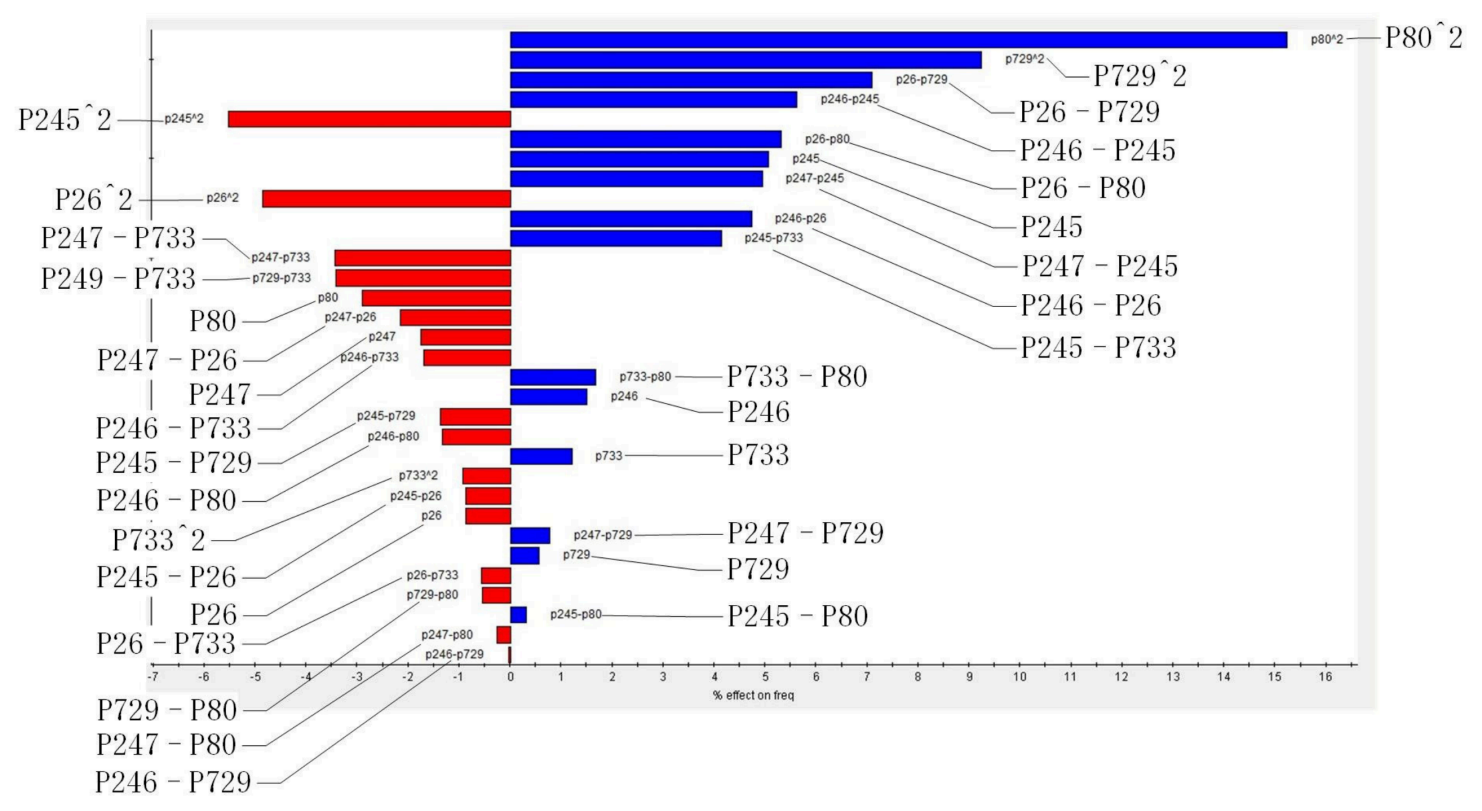
Pareto result of frequency.

Based on the results from 1000 sets of simulation data, the Pareto results, namely the sensitivity analysis results between input parameters and output parameters, are shown in Figure 10 and Figure 11 below.

As can be seen from Figure 10, P80, P246, P26, P729, and P245 exhibit high sensitivity to compliance. Among these, the first four items are negatively correlated parameters, while the last one is positively correlated. Since compliance is inversely proportional to the primary mirror’s stiffness, to reduce compliance, it is necessary to either increase the negatively correlated parameters or decrease the positively correlated parameter.

Similarly, in Figure 11, P80 and P729 show high sensitivity to frequency, and both are positively correlated parameters. To increase the frequency, the positively correlated parameters need to be increased.

Based on the analysis of each parameter’s characteristics, P80, P729, and P246 are ultimately selected for further simulation analysis within narrowed numerical ranges.

#### 3.2.3. Optimal Result

**Figure 12:**
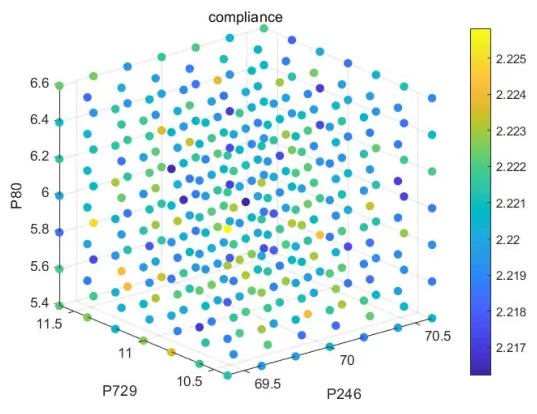
Compliance scatter plot.

**Figure 13:**
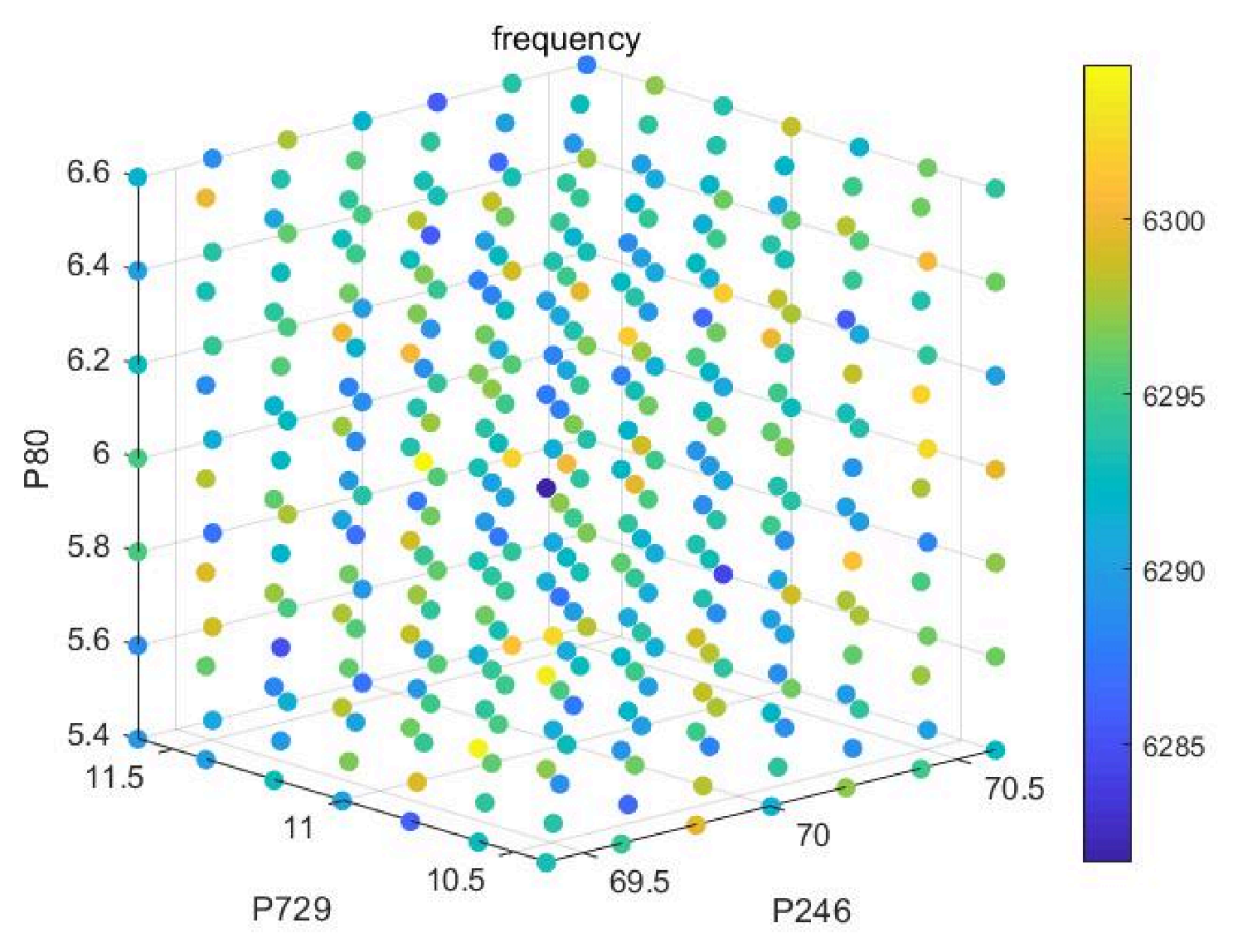
Frequency scatter plot.

Through simulation optimization of the above three parameters with respect to compliance and frequency, functional relationships can be obtained. The relationship between the three parameters and compliance is presented in Figure 12, while the relationship with the fundamental frequency is shown in Figure 13. The minimum value needs to be identified from the compliance scatter plot and the maximum value from the fundamental frequency scatter plot. Based on the constraints in this optimization, including structural stiffness, fundamental frequency, and weight, the final selected values are listed below: P246 = 70 mm, P729 = 11 mm, and P80 = 6 mm.

**Figure 14:**
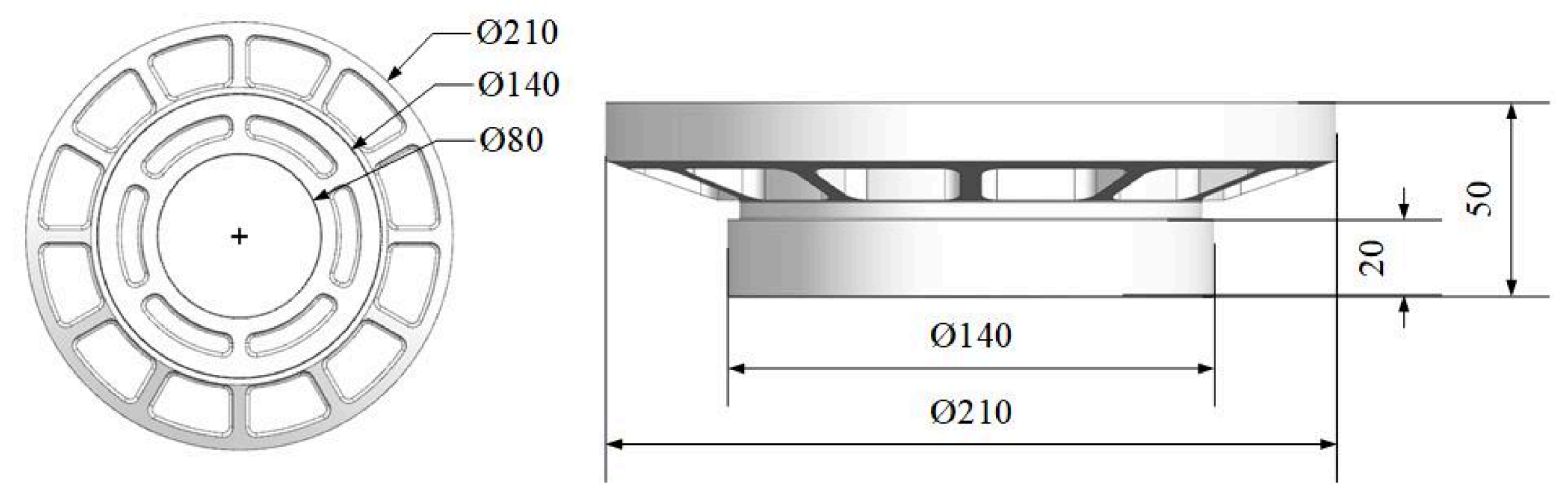
Detailed dimensions of the mirror.

The dimensions of the mirror with the final optimal parameters are depicted in Figure 14.

### 3.3. Static and Modal Analysis

**Figure 15:**
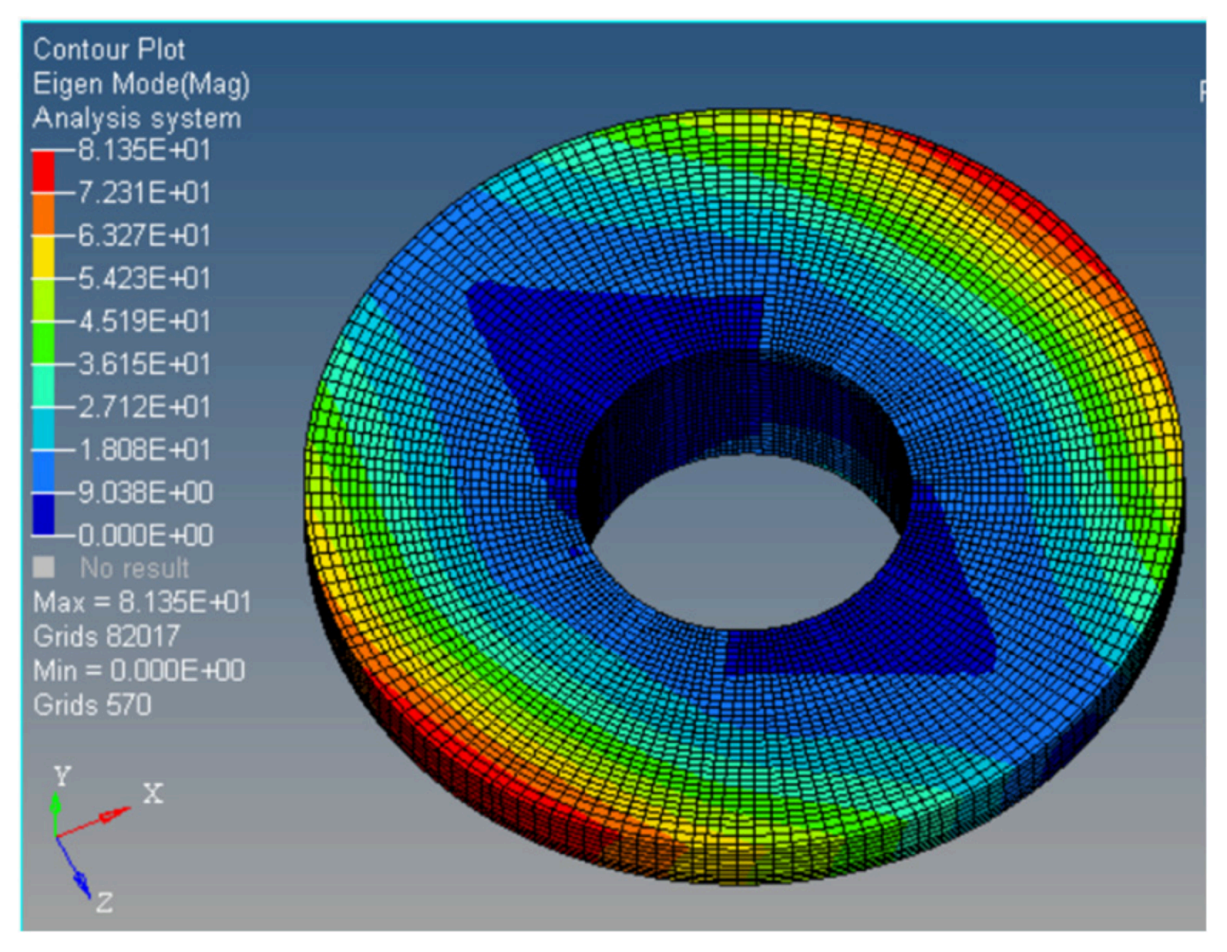
Fundamental frequency of the mirror.

After completing the topological optimization and dimensional optimization, the structure and detailed parameters of the primary mirror are finally determined. An analysis of the fundamental frequency of the primary mirror is conducted, and the analysis result is as shown in Figure 15. According to the results, the fundamental frequency of the primary mirror is 4500 Hz, which is different from the result obtained in the dimensional optimization because the boundary conditions and loads are much closer to the actual situation.

Furthermore, it needs to be ensured that the surface figure of the mirror under gravity is sufficiently small and it will not influence the actual working performance of the imager in space. Therefore, the displacements of the primary mirror under 1 g of gravitational acceleration in three directions are analyzed, and the corresponding surface figure variations are calculated based on the optomechanical coupling analysis method. The analysis results in the three directions are as shown in Table 3.

Summarizing the above analysis results, the displacement and surface figure data are as shown in Table 4. According to the analysis results, since the optical axis is aligned with the Y-direction, the surface figure variation in the Y-direction is relatively significant. That is because when the direction of gravity is parallel to the optical axis of the mirror, the influence of gravity on the deformation of the mirror is the greatest since the bending moment induced by the gravity at the edge of the mirror is the greatest. The X and Z directions are perpendicular to the optical axis, so the surface figure variations in these directions are relatively small.

## 4. Integration and Test

### 4.1. Integration and Test of Primary Mirror Assembly

**Figure 16:**
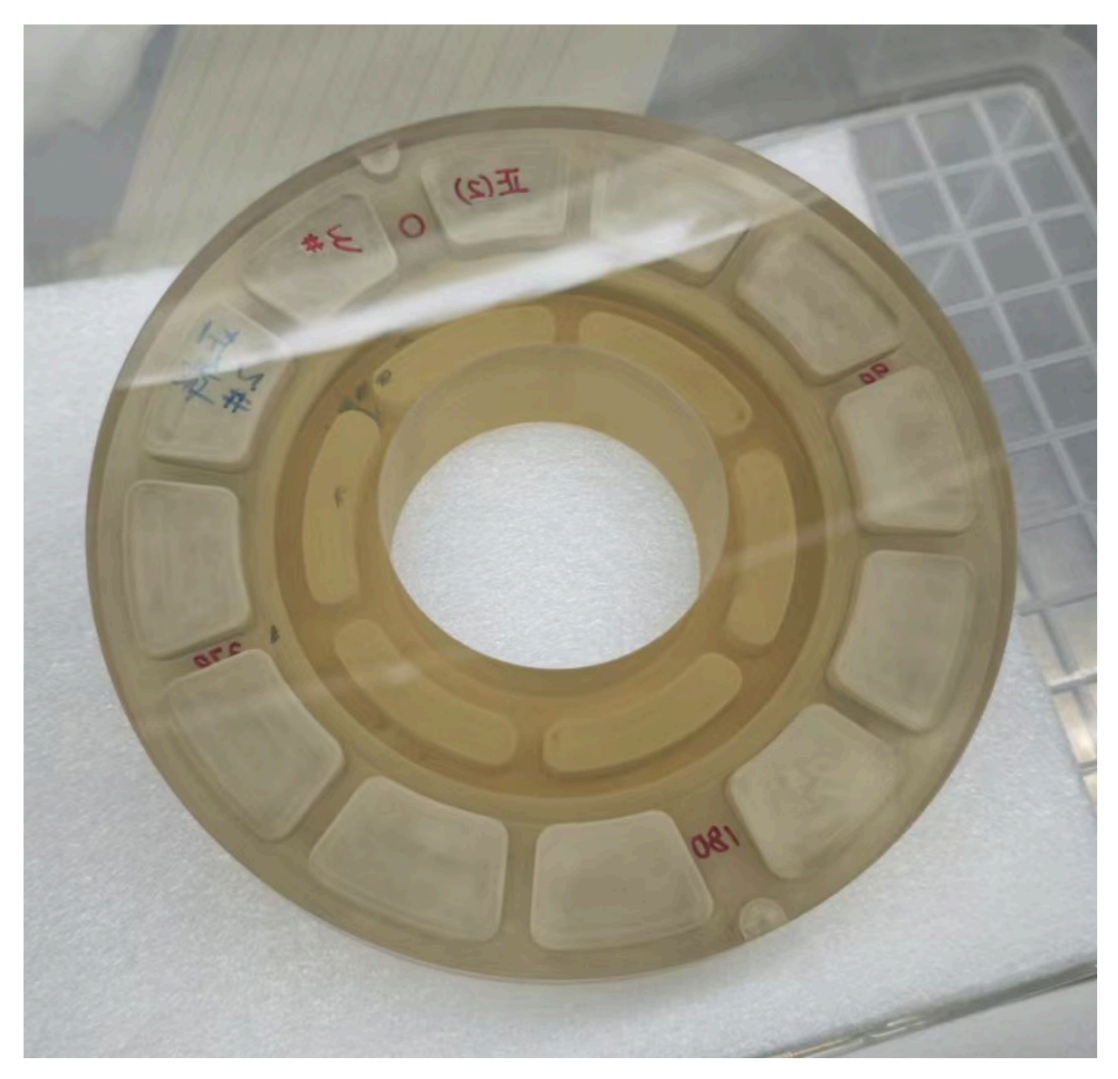
Primary mirror structure.

**Figure 17:**
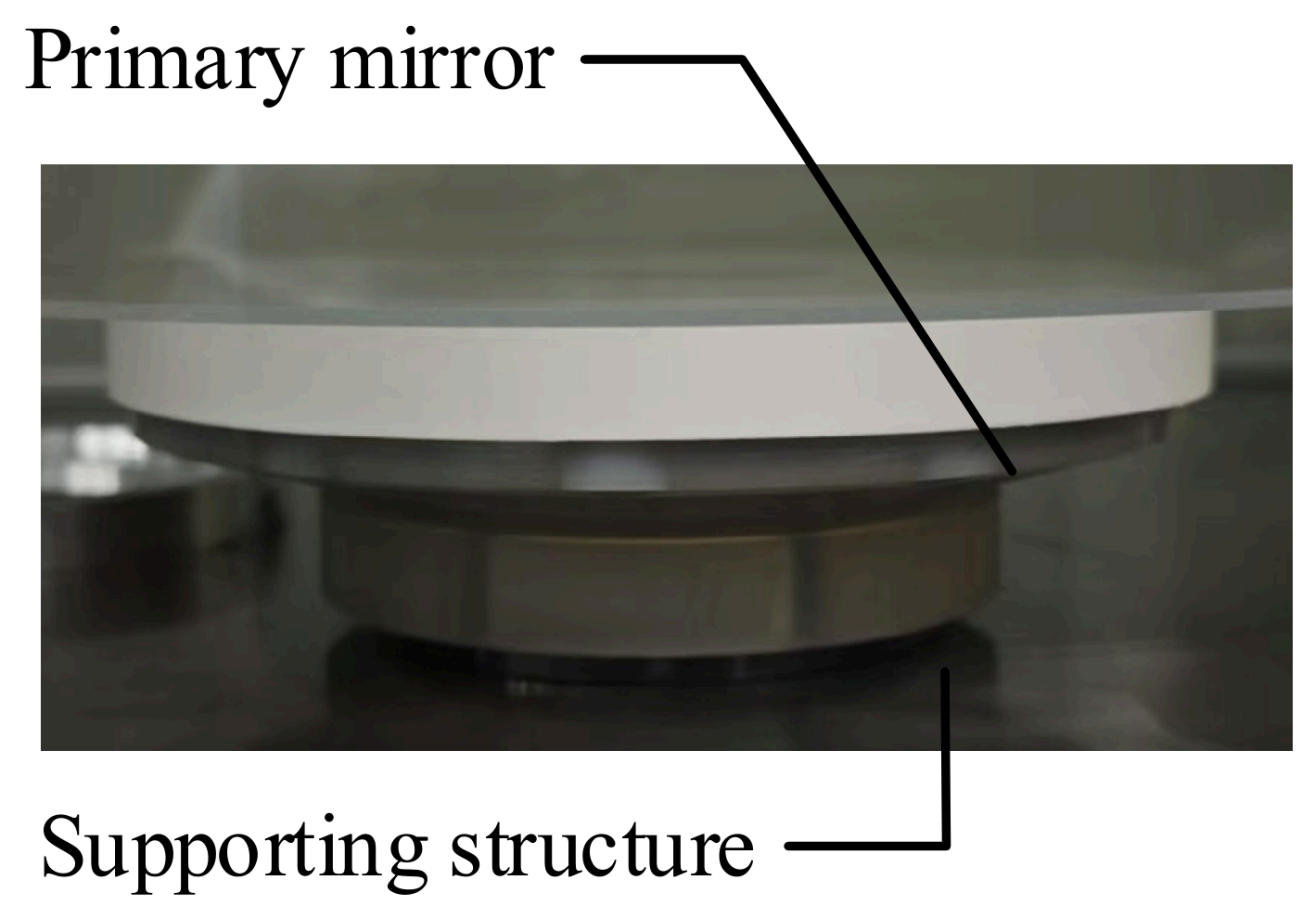
Primary mirror assembly.

Based on the above analysis results, the design parameters of the primary mirror have been determined, and the performance parameters such as the surface figure and fundamental frequency of the primary mirror under this structural form have been clarified. Next, the primary mirror and its supporting structure start to be manufactured. The primary mirror is fabricated using Zerodur, and the supporting structure is made of invar. The primary mirror and assembly are as shown in Figure 16 and Figure 17.

**Figure 18:**
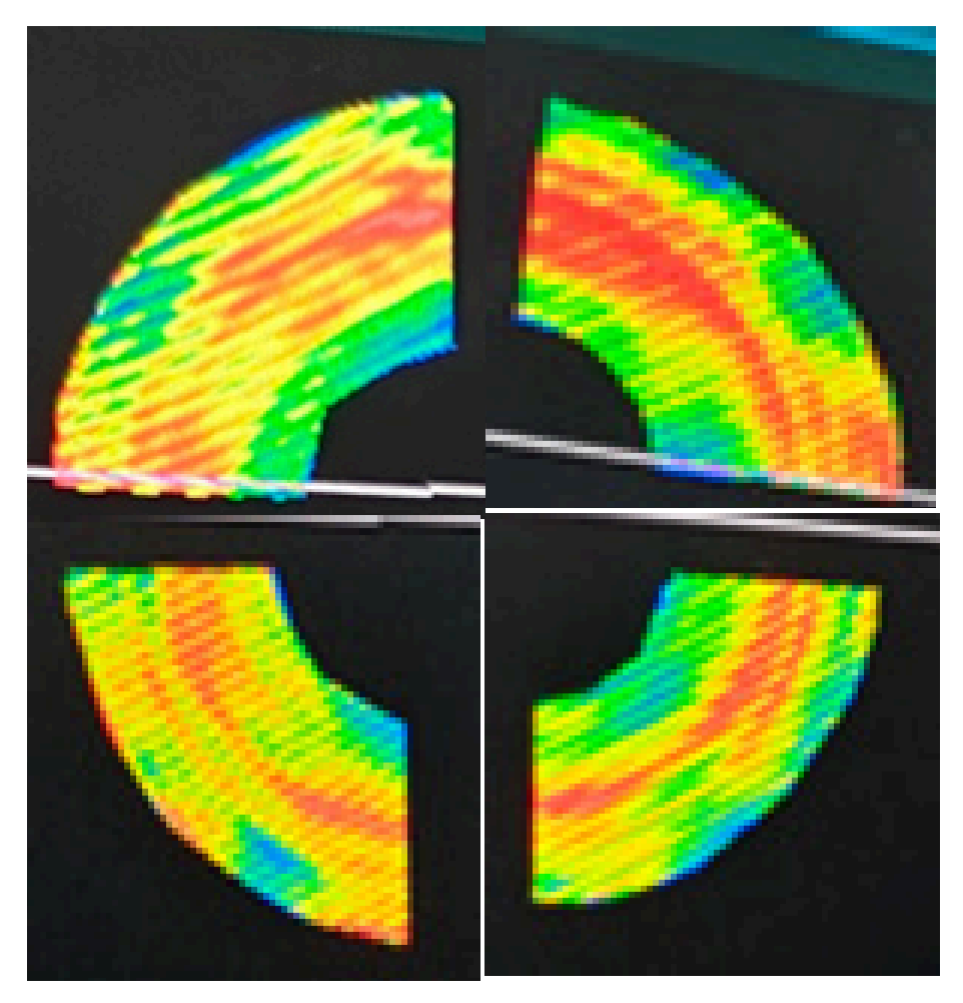
Surface figure result.

To ensure that the surface figure of the primary mirror meets the requirements before and after bonding with the structure, surface figure measurements of the primary mirror are conducted at various stages, respectively. The surface figure result is as shown in Figure 18 and the data comparison at each stage is as shown in Table 5.

When measurement errors and repeatability are taken into consideration, it can be concluded that there is no significant change in the surface figure of the primary mirror before and after bonding, and the bonding process does not introduce external stress. Also, multiple measuring angles are adopted and the deviation of results are less than 0.002λ, which demonstrates that the surface figure of the mirror cannot be influenced by gravity.

### 4.2. Performance Test of Optical System

**Figure 19:**
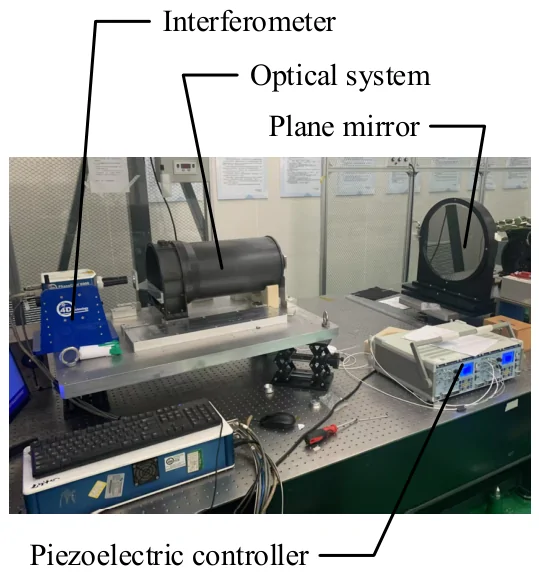
Wavefront aberration test.

After the bonding of the primary mirror assembly is completed, a wavefront aberration test is conducted on the optical system to ensure that the wavefront of the optical system meets the specification requirements. The test state of the system’s wavefront error is as shown in Figure 19. The final system wavefront aberration is 0.08λ, which is less than 0.1λ.

## 5. Conclusions

This paper presents a research methodology for optimizing large-aperture optical primary mirrors, achieving significant performance improvements through comprehensive topology and dimensional optimization. First, an optomechanical coupling-based finite element data processing algorithm is proposed to automatically decompose displacement results from finite element analysis, extracting rigid-body displacement and surface distortion. Second, topology optimization is performed on the mirror, and a dimensional optimization simulation workflow is established in ISIGHT to determine the key dimensional parameters of the mirror. Next, based on the detailed design results, surface shape and modal simulations are conducted to evaluate the mirror’s performance. Finally, experimental validation is carried out, including surface shape testing before and after mirror bonding and wavefront error measurement of the primary–secondary mirror optical system. The test results show that the mirror surface RMS is 0.016λ before bonding and 0.022λ after bonding, indicating that the support structure does not introduce significant stress. The system wavefront error is 0.08λ, surpassing the design threshold of 0.1λ, demonstrating the engineering feasibility of the optomechanical coupling optimization method.

The proposed parametric modeling, sensitivity analysis, and multi-platform collaborative optimization workflow can be extended to the structural design of other optical space systems. Future work may further integrate thermo-mechanical coupling analysis to enhance in-orbit environmental adaptability.

## Figures and Tables

**Table 1:** Material properties.

Parameters	Zerodur	Invar
Elasticity modulus (GPa)	92	141
Poisson ratio	0.3	0.3
Coefficient of thermal expansion (10^−6^/°C)	0.05	0.3
Density (10^3^ kg/m^3^)	2.5	8.1

**Table 2:** Parameters in dataflow.

Input Parameters	Parameter Meaning
P26	Depth of lightening pocket
P80	Width of radial rib
P245	Inner radius of lightening pocket
P246	Radius of boding surface
P247	Outer radius of lightening pocket
P729	Mirror edge thickness
P733	Height of bonding cylindrical surface
**Output parameters**	**Parameter meaning**
Compliance	Stiffness of the mirror structure
Frequency	Fundamental frequency of the mirror structure

**Table 3:** Displacement and surface figure.

	Displacement	Surface Figure
X	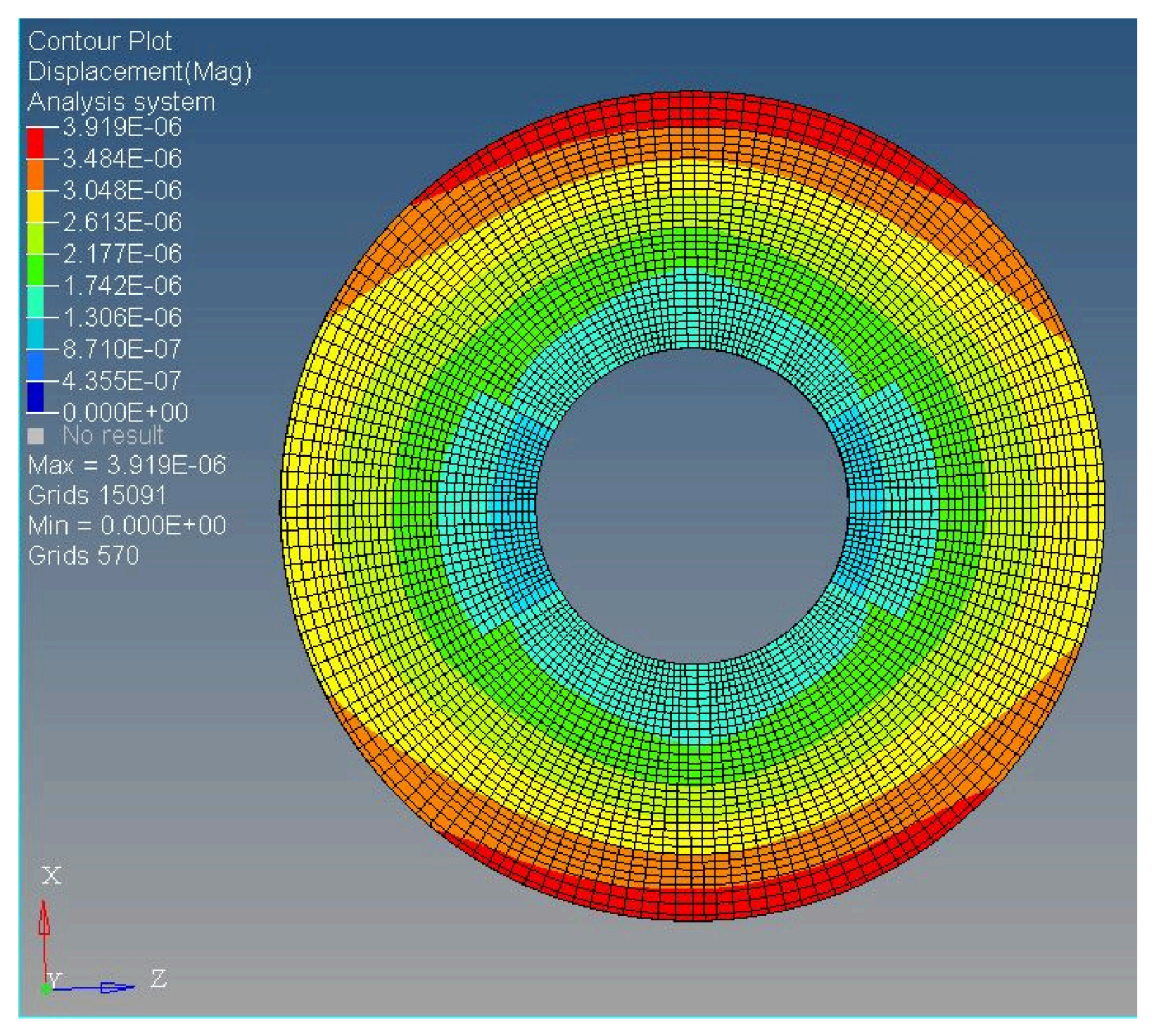	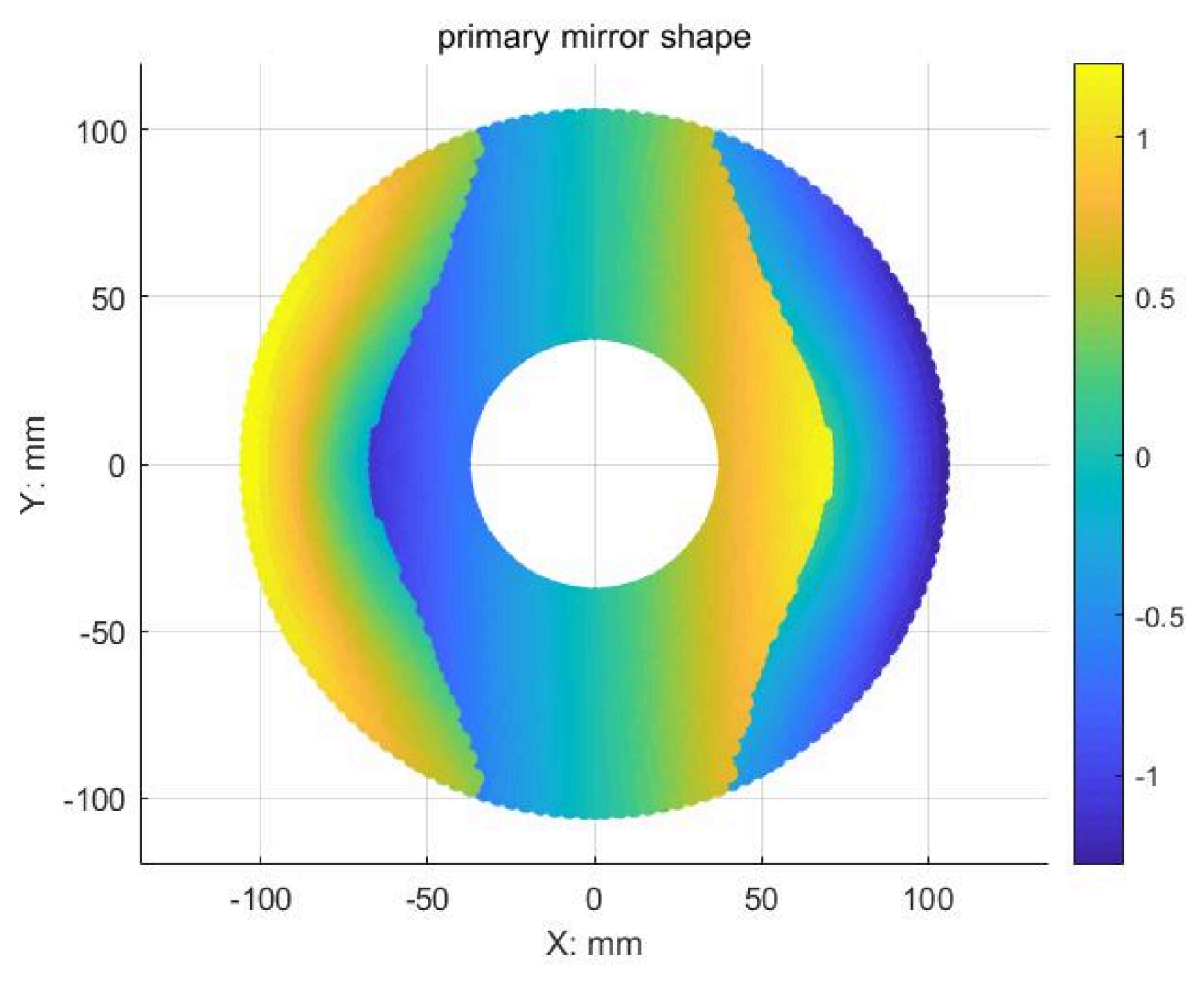
Y	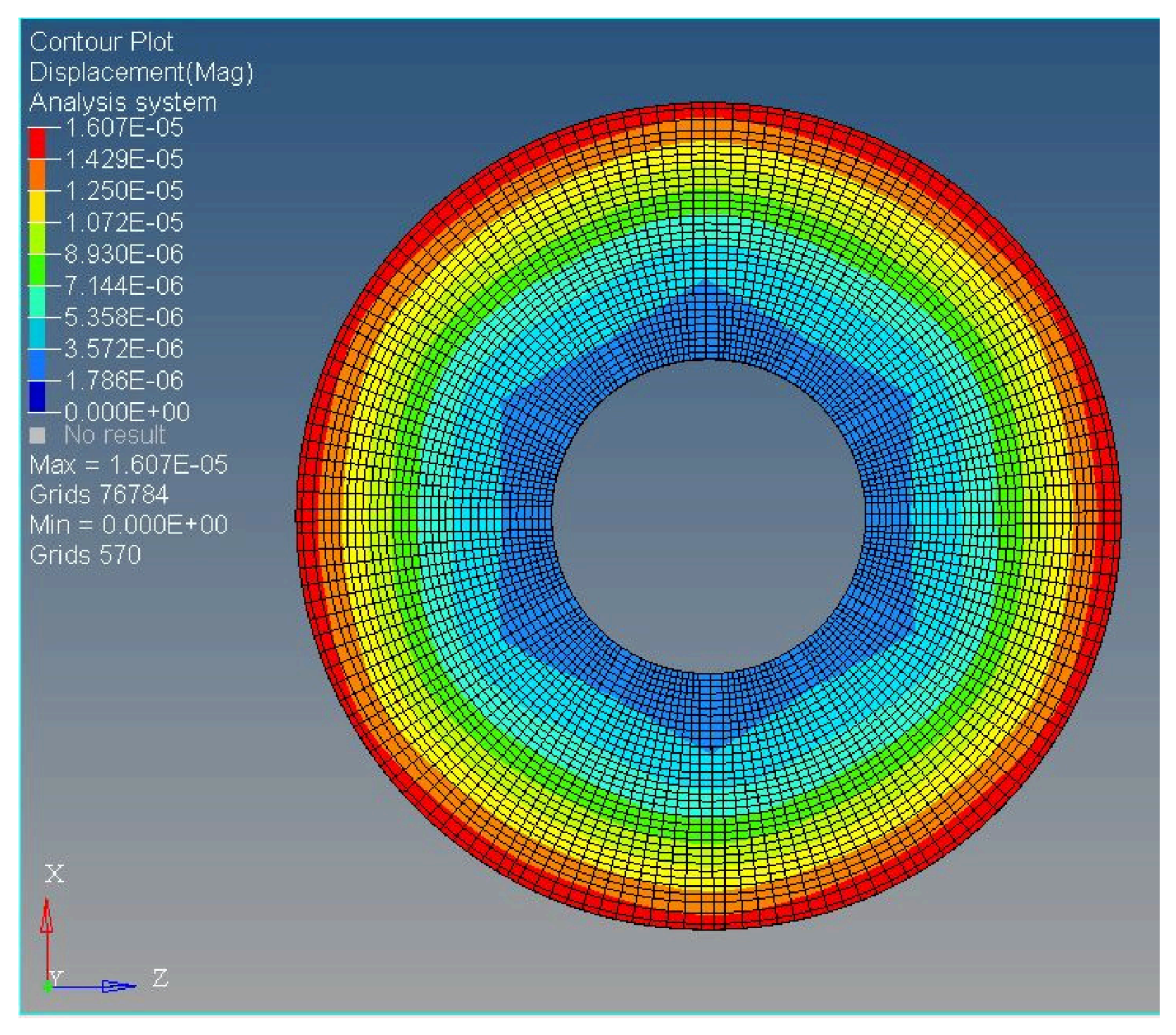	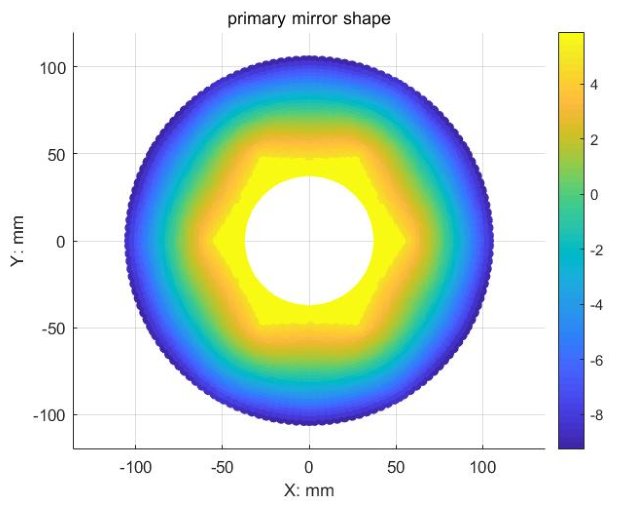
Z	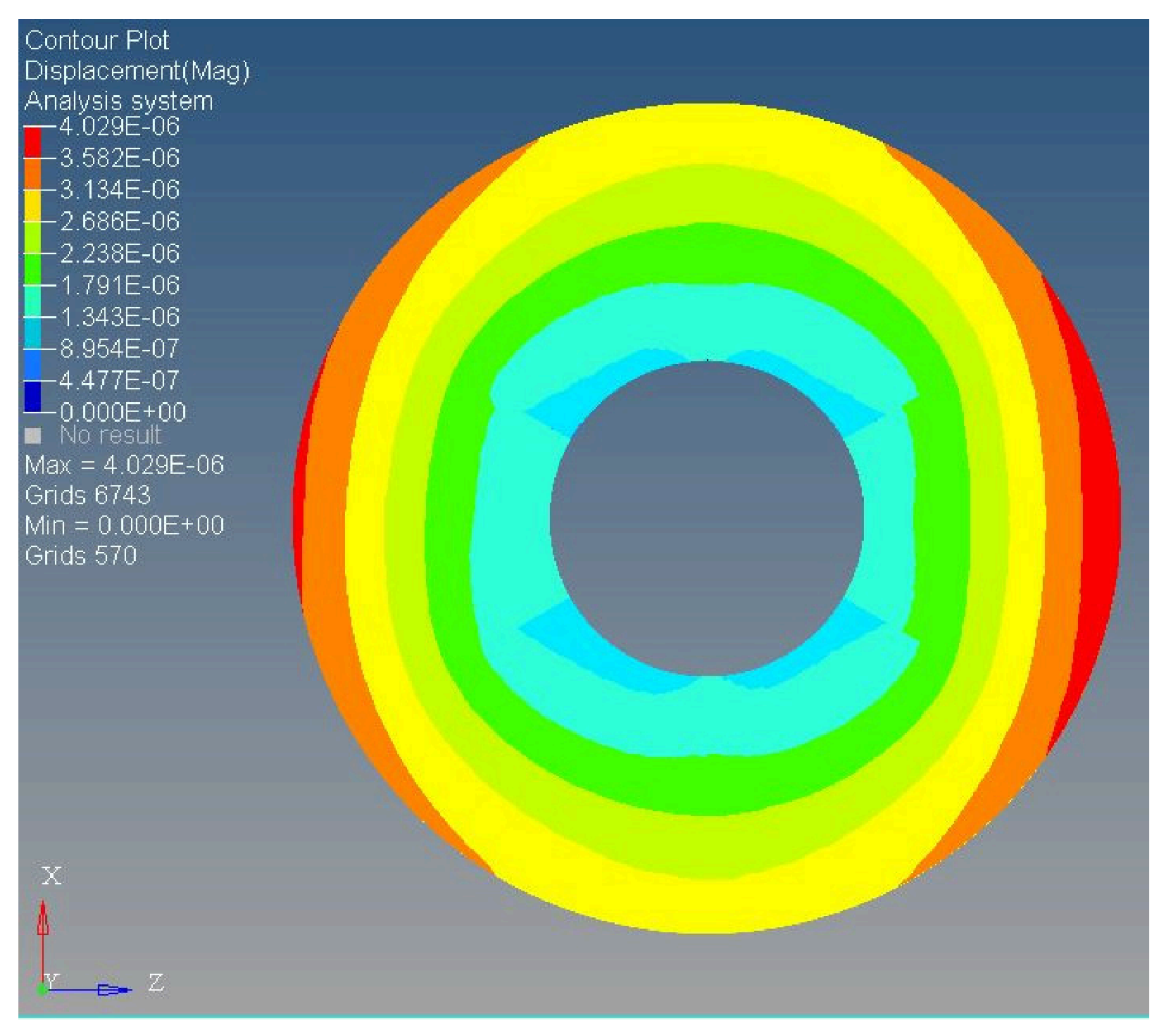	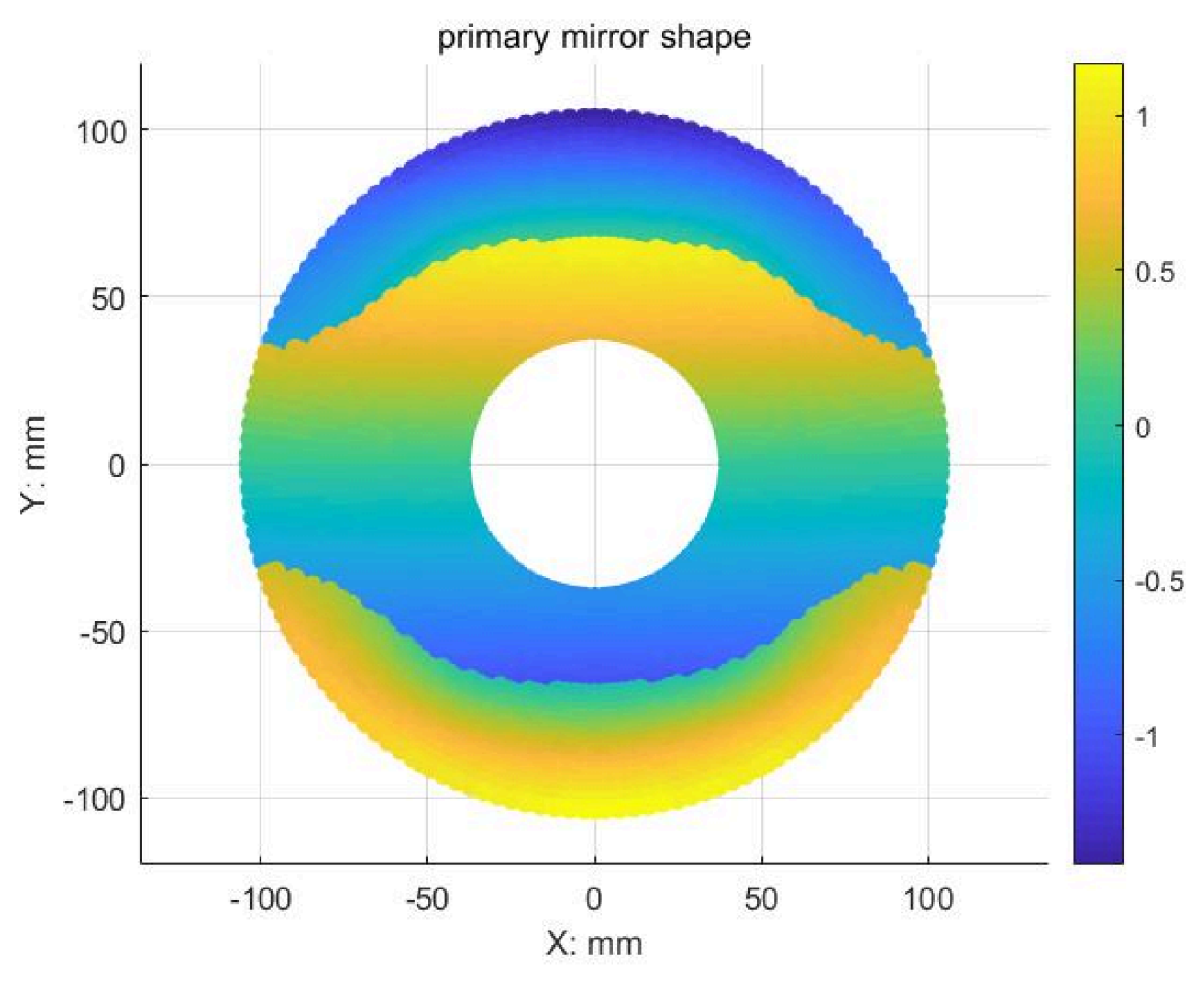

**Table 4:** Displacement and surface figure data.

	Displacement (nm)	Surface Figure PV (nm)	Surface Figure RMS (nm)
X	3.9	2.5	0.6
Y	16	15.2	4.5
Z	4	2.6	0.6

**Table 5:** Surface figure.

	Surface Figure RMS (nm)
Before bonding with structure	0.016λ (λ = 632.8 nm)
After bonding with structure	0.022λ (λ = 632.8 nm)

## Data Availability

The datasets and computer codes used during the current study are available from the corresponding author on reasonable request.
